# Li_2_FeCl_4_ as a Cost-Effective and Durable Cathode for Solid-State Li-Ion Batteries

**DOI:** 10.1021/acsenergylett.4c02376

**Published:** 2024-10-21

**Authors:** Zhantao Liu, Guangxing Zhang, Jakub Pepas, Yifan Ma, Hailong Chen

**Affiliations:** † George W. Woodruff School of Mechanical Engineering, Georgia Institute of Technology, Atlanta, Georgia 30332, United States

## Abstract

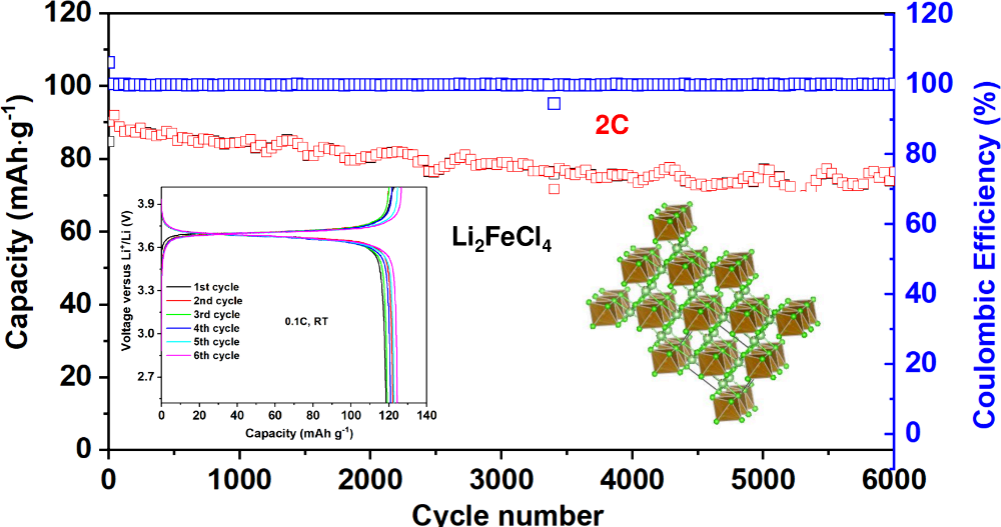

Low-cost cathode materials with high energy density and good rate performance are critical for the development of next-generation solid-state Li-ion batteries (SSLIBs). Here, we report Li_2_FeCl_4_ as a cathode material for SSLIBs with highly reversible Li intercalation and deintercalation, a high operation voltage of 3.7 V vs Li^+^/Li, good rate capability, and good cycling stability with an 86% capacity retention after 6000 cycles. Operando synchrotron XRD reveals that the phase evolution of Li_2_FeCl_4_ during charge–discharge cycling involves both solid-solution and two-phase reactions, which maintains a very stable framework during Li insertion and extraction.

A significant reduction of manufacturing cost is critical to the applications of lithium-ion batteries (LIBs) in electric vehicles and grid-scale energy storage.^1^ In current commercial LIBs, the major cost is associated with the cathode materials (e.g., LiCoO_2_,^2^ LiNi_1–*x*–*y*_Mn_*x*_Co_*y*_O_2_,^3−6^ and LiMn_2_O_4_^7^), as they contain semirare metal elements such as Co, Ni, and Mn serving as the redox-active centers to accommodate Li^+^ insertion and extraction during the charge–discharge cycles. To reduce the cost of LIBs, it is critical to develop new cathodes with naturally abundant, low-cost redox-active elements. Iron (Fe) is an attractive redox-active element in that regard due to its high abundance. However, lithium iron oxide (LiFeO_2_^8^) has a low redox potential (∼2.5 V vs Li^+^/Li) and very poor cycling, and thus is not used. Incorporating Fe into highly inducive PO_4_^3–^ polyanion frameworks raises the redox potentials of Fe^3+^/Fe^2+^ to ∼3.4 V and results in a successful cathode LiFePO_4_.^9^ Yet, the manufacturing process of LiFePO_4_ is quite expensive, which consumes the cost advantage of the raw materials. Iron-fluoride-based cathodes are extensively investigated.^10−12^ However, due to the high electronegativity of F, fluoride cathodes commonly undergo conversion reactions and suffer from high voltage hysteresis caused by the sluggish kinetics.^10^ On the other hand, the use of iron chlorides as cathodes was long overlooked in conventional LIB configurations owing to their high solubility in organic liquid electrolytes.^13^ However, recent progress in solid-state LIBs (SSLIBs) and solid electrolytes is opening up new opportunities to explore or revisit electrode materials that previously did not work with liquid electrolytes, such as FeCl_3_.^14^

Lithium iron chloride Li_2_FeCl_4_ was investigated as a solid electrolyte in the early 1980s.^15−17^ It crystallizes in an inverse spinel structure at high temperature and transforms into an orthorhombic phase at room temperature with an ionic conductivity of ∼10^–5^ S/cm^18^, which is comparable to that of LiFePO_4_.^19^ Its intrinsic high ionic conductivity makes it a potential high-power cathode material. Tanibata et al.^20^ and Vinado et al.^21^ used Li_2_FeCl_4_ as a cathode material in SSLIBs cycled at low current rates, but the rate performance was poor and the cycling life was unsatisfactory. Here, we report the successful realization of stable long-term cycling and good rate performance of Li_2_FeCl_4_ as the cathode in SSLIBs employing chloride solid electrolytes and the new understanding of the phase evolution of Li_2_FeCl_4_ upon Li^+^ insertion/extraction, supported by operando synchrotron X-ray diffraction (SXRD) characterizations. Our results show that Li_2_FeCl_4_ has a complicated but stable and reversible solid-solution and a two-phase reaction pathway. This mechanistic insight provides guidelines for designing halide cathodes with high rate performance and long cycling life for SSLIBs in the future.

Li_2_FeCl_4_ was synthesized via a mechanochemical method, followed by an annealing at 400 °C and 5 h. As shown in Figure 1a, the ionic conductivity of the as-synthesized Li_2_FeCl_4_ was measured to be 1 × 10^–5^ S/cm at room temperature (RT) through electrochemical impedance spectroscopy (EIS), a value consistent with previous reports.^16,22^ The electronic conductivity of the as-synthesized Li_2_FeCl_4_ was measured to be 2 × 10^–7^ S/cm through a direct current (DC) polarization test, and the result is shown in Figure S1. SXRD was used to investigate the crystal structure of as-synthesized Li_2_FeCl_4_, and the Rietveld refinement results are shown in Figure 1b and Table S1. In previous literature reports, two different structure models are proposed to describe Li_2_FeCl_4_: Kanno et al. proposed that Li_2_FeCl_4_ crystallizes in an ordered spinel superstructure within an *Imma* space group^16^ and Lutz et al. suggested that Li_2_FeCl_4_ is a mixture of a SnMn_2_S_4_-type NaCl superstructure in a *Cmmm* space group and Li_6_FeCl_8_ in a cubic phase.^18^ The *Imma* phase is a special case of the more general *Cmmm* phase, with additional Li sites being partially occupied. In Rietveld refinements against the XRD pattern of our sample, solely using either the *Imma* or *Cmmm* structure model yielded poor *R*_wp_ of 11.81% and 11.74%, respectively (Figures S2 and S3). A substantially improved *R*_wp_ of 7.76% was achieved in a two-phase refinement using both structures, indicating that the as-synthesized Li_2_FeCl_4_ mainly crystallized in the SnMn_2_S_4_-type NaCl superstructure with a small portion of the ordered spinel superstructure. Their structures are shown in Figures 1c and 1d, respectively. Both structures adopt ABCABC. . . type Cl^–^ anion stacking and share similar Fe sublattices. In both cases, Fe ions reside in octahedral sites, and the FeCl_6_ octahedra are connected via edge-sharing to form a chain. Both structures can be viewed as layered. Within the Fe-containing layer, besides the octahedral sites occupied by Fe^2+^, the other octahedral sites (e.g., 4f sites in the *Cmmm* structure and 4b sites in the *Imma* structure) are fully occupied by Li^+^ ions. The Li distributions in the Fe-containing layers in the two structures are slightly different. In the *Imma* structure, Li ions reside in both octahedral and tetrahedral sites, while in the *Cmmm* structure, Li ions exclusively reside in octahedral sites. Figure 1e shows the migration pathways for Li^+^ ions in the major phase, i.e., the Li_2_FeCl_4_ in *Cmmm* structure, analyzed by the bond valence site energy (BVSE) method.^23,24^ The BVSE results suggest that Li^+^ ions are metastable at the vacant octahedral sites (“Oct”, 2b, at (0.5, 0, 0)). The empty tetrahedral sites (“Tet”) connect the fully occupied Li sites and the empty octahedral sites in a face-shared manner, forming 1D [Li–Tet–Oct–Tet–Li] zigzag Li diffusion pathways along different directions. These 1D pathways further interconnect with one another to form a 3D transport network. The calculated activation energy by the BVSE method is shown in Figure 1f, suggesting the highest migration barrier is associated with Li^+^ hopping to the metastable Tet sites. Figure S4 shows the in situ XRD of the as-synthesized Li_2_FeCl_4_. It shows good thermal stability, and no decomposition is observed within the temperature range from room temperature to 400 °C.

**Figure 1:**
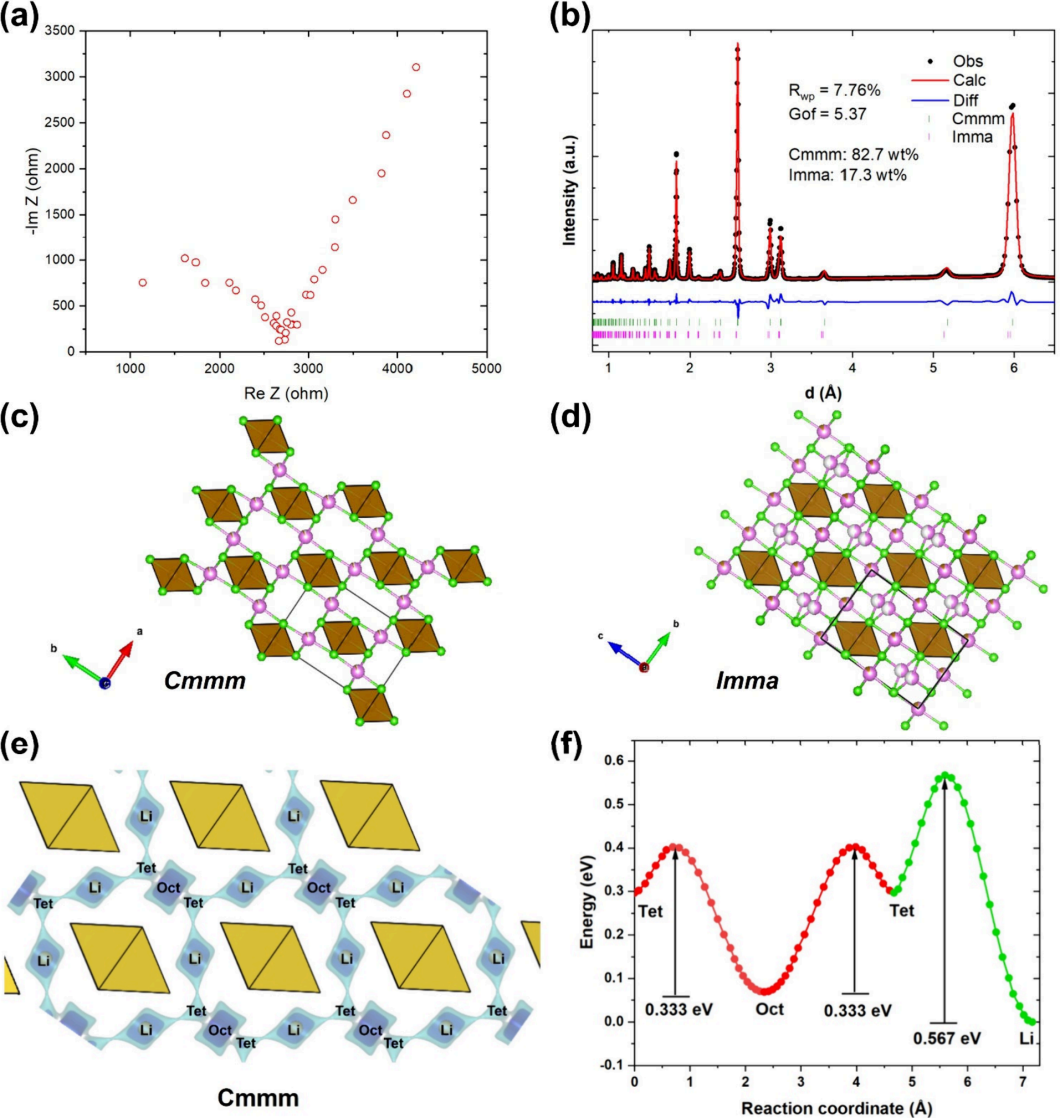
Li^+^conduction path in Li_2_FeCl_4_. (a) Nyquist plots of Li_2_FeCl_4_ pellet measured at room temperature. (b) Rietveld refinement against synchrotron powder X-ray diffraction pattern of Li_2_FeCl_4_. Diffraction data are shown in black dots, the calculated pattern is shown as a red curve, and the difference curve is shown in blue. The Bragg reflection positions of the two phases are shown as olive and pink bars, respectively. (c and d) Li_2_FeCl_4_ structures with *Cmmm* (c) and *Imma* (d) space groups. Fe is in brown, Li is in pink, and Cl is in green. (e) Li^+^ potential map calculated by BVSE superimposed with the crystal structure of Li_2_FeCl_4_ in the *Cmmm* space group. (f) The energy profile of the migration pathways in Li_2_FeCl_4_ calculated based on the *Cmmm* structure.

The cycling performance of Li_2_FeCl_4_ in SSLIBs was assessed employing Li_2.75_In_0.75_Zr_0.25_Cl_6_^25^ and Li_3_YCl_3_Br_3_^26^ as the electrolytes and In–Li alloy as the anode. Figure 2a shows the typical charge–discharge voltage profiles of Li_2_FeCl_4_ in the initial six cycles at a 0.1 C rate (1 C = 126 mAh/g) at RT. The loading of Li_2_FeCl_4_ is 4.34 mg/cm^2^. During the initial charge, Li_2_FeCl_4_ exhibits a main plateau at ∼3.7 V vs Li^+^/Li with a specific capacity of 130 mAh/g, slightly over the theoretical capacity (126 mAh/g) calculated based on the Fe^3+^/Fe^2+^ redox reaction. Meanwhile, a minor plateau is observed above 4.05 V, which can be ascribed to the side reaction associated with Cl^–^ oxidation^27^ and is responsible for the part of the capacity exceeding the theoretical value. Thus, the charge cutoff voltage was adjusted to 4.02 V vs Li^+^/Li to avoid the side reaction. In the following cycles, the discharge capacity increased gradually and reached 124 mAh/g in the sixth cycle. Figure 2b shows the corresponding d*Q*/d*V* curves between 2.52 and 4.02 V vs Li^+^/Li. The d*Q*/d*V* curves show a primary pair of redox peaks centered at 3.71 V (anodic) and 3.68 V (cathodic) vs Li^+^/Li, which are higher than that of LiFePO_4_ (3.4 V vs Li^+^/Li) but lower than LiCoO_2_ (4 V vs Li^+^/Li) and LiNi_0.8_Co_0.1_Mn_0.1_O_2_ (3.7–3.8 V vs Li^+^/Li).^28,29^Figures 2c and 2d show the rate performance of Li_2_FeCl_4_ cells at RT. Because of its intrinsic high ionic conductivity, Li_2_FeCl_4_ exhibits high capacities of 117, 109, 102, 90, and 65 mAh/g at the rates of 0.2, 0.5, 1, 2, and 5 C, respectively. It is striking that at the high rate of 5 C, which translates to a 12 min fast discharge, the cell still can deliver 52% of the theoretical capacity, which outperforms most existing oxide cathodes in solid-state batteries. Following the high-rate cycling, the cell was subjected to a long-term cycling test at 2 C for 6000 cycles. The discharge capacity retains 86% after 6000 cycles, demonstrating the excellent cycling stability of the Li_2_FeCl_4_. Figure S5 shows the cycling performance of a Li_2_FeCl_4_ cell with higher cathode mass loading (17.6 mg/cm^2^ Li_2_FeCl_4_). The cell was activated at the initial four cycles at 0.1 C. Even with this high loading, the Li_2_FeCl_4_ cell still delivers a high specific capacity of 116 mAh/g, (92% of the theoretical capacity), resulting in an areal capacity of 2 mAh/cm^2^ at 0.1 C at RT. Subsequently, the high-loading Li_2_FeCl_4_ cell was cycled at 0.25 C, achieving a capacity of up to 110 mAh/g with a capacity retention of 81% over 720 cycles.

**Figure 2:**
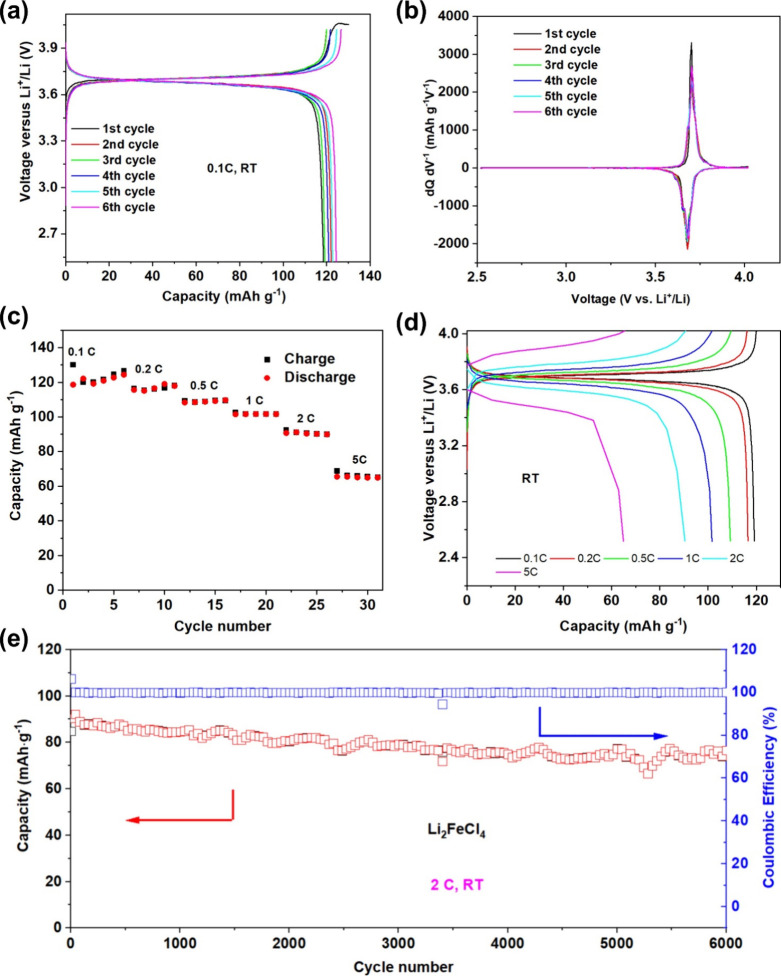
Rate performance of the Li_2_FeCl_4_ cathode in solid cells at RT. (a) Charge/discharge curves of Li_2_FeCl_4_ in the initial six cycles at 0.1 C and RT and (b) corresponding d*Q*/d*V* curves. (c) Charge/discharge capacity as a function of cycle number at different C rates at RT and (d) corresponding charge/discharge curves. (e) Long-term cycling of the Li_2_FeCl_4_ solid cell at 2 C for 6000 cycles at RT.

To gain more insight into the phase evolution of Li_2_FeCl_4_ during the charge–discharge cycling, an operando SXRD experiment was performed. A solid-state cell comprising a composite cathode of Li_2_FeCl_4_/ball-milled Li_3_YCl_6_/carbon (with a wt % ratio of 55:40:5), a separator of ball-milled Li_3_YCl_6_ and an In–Li anode was assembled and then cycled under a 0.1 C rate at RT between 2.52 and 4.39 V vs Li^+^/Li. Figure 3a shows a schematic of the operando SXRD setup. The monochromatic synchrotron X-ray transmits through the cell along the radius direction, and the diffraction data is collected in real time as the cell is cycled. The axial thickness of the solid-state cell is measured to be ∼650 μm with a cathode thickness of ∼100 μm. The X-ray beam size along the vertical direction is ∼100 μm, ensuring only cathode and separator layers are scanned during the test. A wider beam size along the horizontal direction is used (∼600 μm) to obtain good statistics and data quality. Figure 3b shows the contour map of the XRD signal of the entire cell in a vertical scan before cycling. The corresponding XRD patterns are shown in Figure S6, where the peaks at ∼5.03° and 5.81° belong to the stainless-steel current collectors, and the peak at 5.13° is from the sealing material.

**Figure 3:**
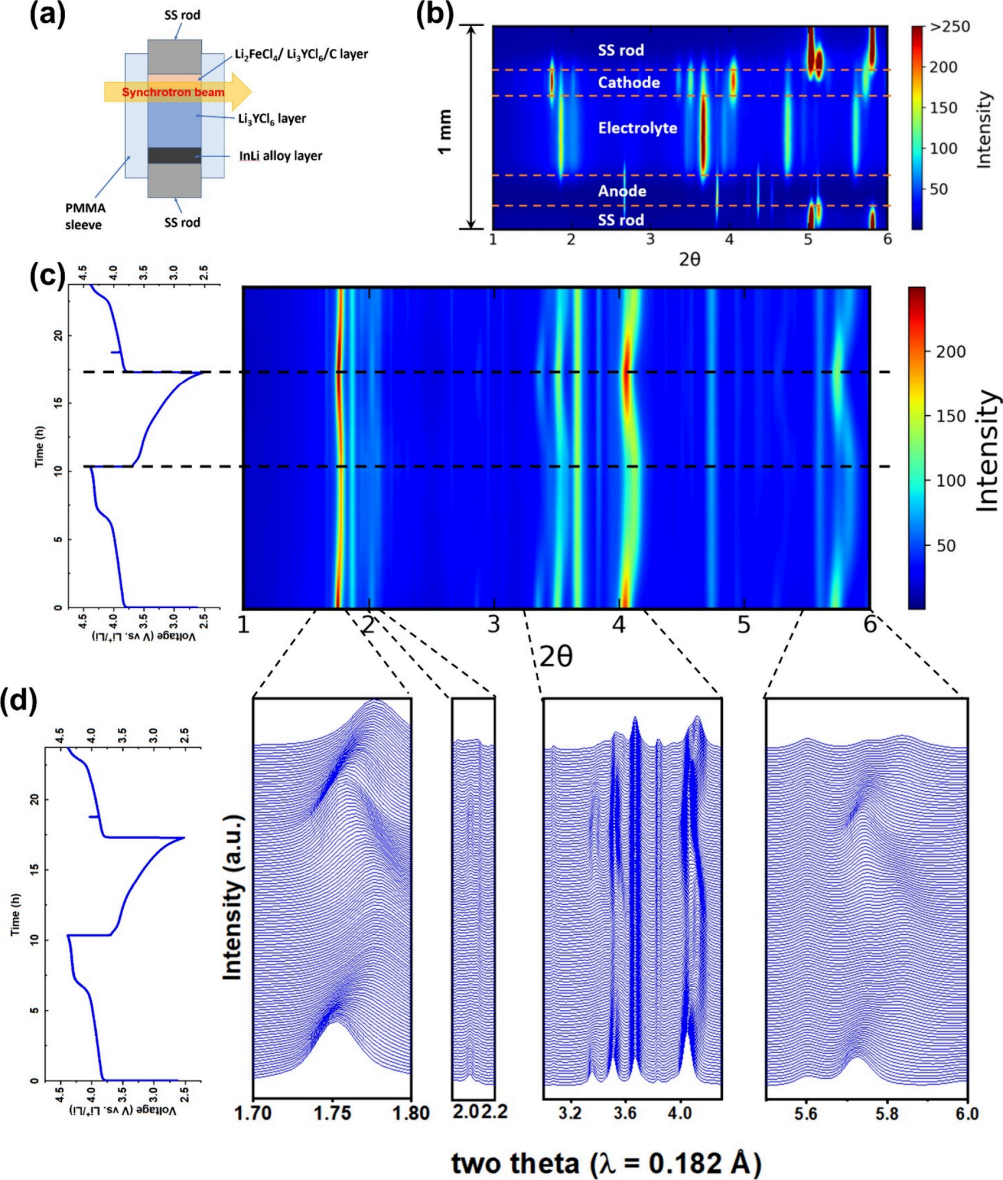
Phase transition of Li_2_FeCl_4_ during charge/discharge test. (a) Schematic illustration of operando synchrotron X-ray diffraction setup. (b) Contour plots between 1° and 6° of the Li_2_FeCl_4_ cell before cycling. (c) Contour plots (right panels) of operando synchrotron X-ray diffraction and corresponding charge/discharge profiles (left panel). (d) Magnified views of synchrotron X-ray diffraction patterns in selected regions.

Figure 3c shows the contour map of the time-resolved operando SXRD patterns solely from the cathode layer between 1° and 6° and the corresponding galvanostatic charge/discharge curves. During the first charge process, a solid solution reaction, followed by a phase transition, is observed. At the early stage of the charge process (*x* ≤ 0.07 of Li_2–*x*_FeCl_4_), the reflections at 1.75°, 3.35°, 3.50°, 4.04°, and 5.73°, which belong to Li_2_FeCl_4_, shift to higher 2θ angles, indicating a solid solution reaction, i.e., the shrinkage of the Li_2_FeCl_4_ unit cell upon Li^+^ extraction (Figure 3d). The intensities of these peaks gradually drop, implying some disordering caused by Li extraction, similar to what is commonly observed in operando XRD of oxide cathodes. As more Li^+^ is extracted from Li_2_FeCl_4_, a set of new peaks emerges at 1.78°, 2.09°, 4.17°, and 5.83°, accompanied by an increase in their intensities as charging progresses, indicating a new phase. The reflection at 3.50° belonging to the solid electrolyte remains intact, as expected. The cell also exhibits an extra plateau above 4.08 V upon further charging, which is consistent with the results shown in Figure 2a, likely due to the oxidation of Cl^–^. Yet, no discernible change in SXRD is observed. In the subsequent discharge process, the SXRD data show a fully reversed phase transition pathway, with the XRD pattern of pristine Li_2_FeCl_4_ being restored upon full lithiation, as shown in Figure S7. The operando SXRD patterns of the second charge show identical trends as those in the first charge, again confirming the high reversibility of the phase evolution.

In summary, highly reversible Li insertion/extraction is realized in Li_2_FeCl_4_ in SSLIBs employing chloride electrolytes. The Li_2_FeCl_4_ SSLIB cells show a high operating voltage of ∼3.7 V vs Li^+^/Li utilizing the Fe^3+^/Fe^2+^ redox, which is higher than that of LiFePO_4_. A high specific capacity of 124 mAh/g, which is almost the theoretical capacity, can be reversibly delivered at 0.1 C at RT. The energy density is estimated at ∼466 Wh/kg, which is lower than that of LiFePO_4_ (∼540 Wh/kg). Benefiting from the high ionic conductivity and highly reversible phase evolution, Li_2_FeCl_4_ shows exceptionally good rate performance under RT among the existing cathode materials for SSLIBs. The highly reversible phase evolution disclosed by the operando synchrotron XRD experiment, though more details such as Li sublattices are to be revealed in future work, explains the outstanding long-term cycling stability of Li_2_FeCl_4_. Compared to the FeCl_3_ cathode recently developed by our group,^9^ Li_2_FeCl_4_ offers another low-cost option for cathodes with sufficient Li sources included. The excellent electrochemical performances of Li_2_FeCl_4_ provide a promising option as a low-cost, high-power cathode for SSLIBs, and its charge storage mechanism offers new opportunities to design and explore new halide-based cathode materials for SSLIBs.

## Experimental Section

### Materials Synthesis

Li_2_FeCl_4_ was synthesized via a mechanochemical method followed by an annealing treatment. LiCl (sigma, 99%) and FeCl_2_ were weighed in a molar ratio of 2:1 and ball-milled at 500 rpm for 5 h in ZrO_2_ jars (50 mL) with ZrO_2_ balls (diameter: 10 mm) in a planetary ball mill (PM 200, Retsch) followed by annealing at 400 °C. All the processes were carried out under Ar atmosphere.

Li_3_YCl_3_Br_3_ and Li_2.75_In_0.75_Zr_0.25_Cl_6_ were prepared by using a similar method. For Li_3_YCl_3_Br_3_ synthesis, LiBr and YCl_3_ were ball-milled at 500 rpm for 5 h followed by sintering at 400 °C. For Li_2.75_In_0.75_Zr_0.25_Cl_6_ synthesis, LiCl, InCl_3_, and ZrCl_4_ were ball-milled at 500 rpm for 5 h followed by sintering at 420 °C.

The Li_3_YCl_6_ was synthesized mechanochemically from LiCl and YCl_3_ at 500 rpm for 5 h.

### Electrochemical Measurements

The ionic conductivity was measured using an electrochemical impedance analyzer (VMP3, Biologic) and a homemade electrochemical cell. Typically, 0.5–1 g of electrolyte powders were cold pressed into pellets with a diameter of 1/2 in. at a pressure of 294 MPa. Two pieces of Al foils were used as current collectors, and the data was collected at varied temperatures in the frequency range of 1 MHz to 1 Hz with an AC amplitude of 50 mV.

The solid-state cells were fabricated with a homemade setup. The composite cathode was made by mixing as-synthesized Li_2_FeCl_4_, Li_2.75_In_0.75_Zr_0.25_Cl_6_, and carbon black in a weight ratio of 55:40:5 in a mortar by hand. InLi alloy was used as the anode. Then, 130 mg of Li_2.75_In_0.75_Zr_0.25_Cl_6_^25^ was pressed at 294 MPa to form a dense pellet within a PMMA sleeve (ID: 1/2 in.). Next, 80 mg of Li_3_YCl_3_Br_3_^26^ was used as protective layer against InLi alloy, which was spread on one side of the Li_2.75_In_0.75_Zr_0.25_Cl_6_ pellet and pressed at 294 MPa, and ∼10 mg of composite cathode was spread on the other side of the Li_2.75_In_0.75_Zr_0.25_Cl_6_ pellet at pressed at the same pressure. Finally, the anode part was fabricated by attaching a piece of Indium foil on the Li_3_YCl_3_Br_3_ side, followed by attaching another piece of lithium metal foil and pressing at ∼50 MPa. Galvanostatic charge/discharge was conducted on the LAND battery test system at room temperature.

### Ex Situ Synchrotron Diffraction Characterization

Synchrotron X-ray diffraction patterns were collected at the synchrotron X-ray source at beamline 17-BM at the Advanced Photon Source (APS) and at the 28ID-2 beamline of the National Synchrotron Light Source II (NSLS II). The photon energies at 17-BM and 28ID-2 are 51.4 and 68.1 keV, respectively. Rietveld refinements against the XRD data were performed with using GSAS II.^30^ The crystal structure was visualized by VESTA.^31^

### Operando Synchrotron Diffraction Characterization

Operando synchrotron diffraction measurements were conducted in a homemade solid-state cell at the 28-ID-B beamline at the National Synchrotron Light Source II (NSLS II) in Brookhaven National Laboratory. In this cell, single Li_3_YCl_6_ layer was employed instead of the Li_2.75_In0_.75_Zr_0.25_Cl_6_/Li_3_YCl_3_Br_3_ bilayer as solid electrolytes. An X-ray transparent tube with an inner diameter of 1/8 in. was employed as sleeve. Then, ∼10 mg of Li_3_YCl_6_ was pressed into a pellet within the tube. The composite cathode was made by mixing as-synthesized Li_2_FeCl_4_, Li_3_YCl_6_, and carbon black in a weight ratio of 55:40:5 in a mortar by hand. In this cell, the cathode mass loading was ∼2 mg. The diffraction patterns were collected in transmission mode. The incident beam size along the vertical direction was narrowed down to ∼100 um, and the vertical length of the cell was scanned layer-by-layer with a step size of 33 μm. The data acquisition time was 60 s.

## Supporting Information Available

Rietveld refinement results, electrochemical testing results, and operando XRD data and tables (PDF)

## References

[ref1] Janek J.; Zeier W. G. A solid future for battery development. Nature Energy 2016, 1, 16141 10.1038/nenergy.2016.141.

[ref2] Mizushima K.; Jones P. C.; Wiseman P. J.; Goodenough J. B. LixCoO2 (0 < x<-1): A new cathode material for batteries of high energy density. Mater. Res. Bull. 1980, 15 (6), 783–789. 10.1016/0025-5408(80)90012-4.

[ref3] Koyama Y.; Tanaka I.; Adachi H.; Makimura Y.; Ohzuku T. Crystal and electronic structures of superstructural Li1-x[Co1/3Ni1/3Mn1/3]O2 (0 ≤ *x* ≤ 1). J. Power Sources 2003, 119–121, 644–648. 10.1016/S0378-7753(03)00194-0.

[ref4] Makimura Y.; Ohzuku T. Lithium insertion material of LiNi1/2Mn1/2O2 for advanced lithium-ion batteries. J. Power Sources 2003, 119–121, 156–160. 10.1016/S0378-7753(03)00170-8.

[ref5] Liu Z.; Yu A.; Lee J. Y. Synthesis and characterization of LiNi1-x-yCoxMnyO2 as the cathode materials of secondary lithium batteries. J. Power Sources 1999, 81–82, 416–419. 10.1016/S0378-7753(99)00221-9.

[ref6] Thackeray M. M.; Amine K. Layered Li-Ni-Mn-Co oxide cathodes. Nature Energy 2021, 6 (9), 933–933. 10.1038/s41560-021-00860-3.

[ref7] Thackeray M. M.; David W. I. F.; Bruce P. G.; Goodenough J. B. Lithium insertion into manganese spinels. Mater. Res. Bull. 1983, 18 (4), 461–472. 10.1016/0025-5408(83)90138-1.

[ref8] Kanno R.; Shirane T.; Kawamoto Y.; Takeda Y.; Takano M.; Ohashi M.; Yamaguchi Y. Synthesis, Structure, and Electrochemical Properties of a New Lithium Iron Oxide, LiFeO2, with a Corrugated Layer Structure. J. Electrochem. Soc. 1996, 143 (8), 2435 10.1149/1.1837027.

[ref9] Padhi A. K.; Nanjundaswamy K. S.; Goodenough J. B. Phospho-olivines as Positive-Electrode Materials for Rechargeable Lithium Batteries. J. Electrochem. Soc. 1997, 144 (4), 1188–1194. 10.1149/1.1837571.

[ref10] Hua X.; Eggeman A. S.; Castillo-Martínez E.; Robert R.; Geddes H. S.; Lu Z.; Pickard C. J.; Meng W.; Wiaderek K. M.; Pereira N.; et al. Revisiting metal fluorides as lithium-ion battery cathodes. Nat. Mater. 2021, 20 (6), 841–850. 10.1038/s41563-020-00893-1. 33479526

[ref11] Fan X.; Hu E.; Ji X.; Zhu Y.; Han F.; Hwang S.; Liu J.; Bak S.; Ma Z.; Gao T.; et al. High energy-density and reversibility of iron fluoride cathode enabled via an intercalation-extrusion reaction. Nat. Commun. 2018, 9 (1), 2324 10.1038/s41467-018-04476-2. 29899467 PMC5998086

[ref12] Li L.; Meng F.; Jin S. High-Capacity Lithium-Ion Battery Conversion Cathodes Based on Iron Fluoride Nanowires and Insights into the Conversion Mechanism. Nano Lett. 2012, 12 (11), 6030–6037. 10.1021/nl303630p. 23106167

[ref13] Wu F.; Yushin G. Conversion cathodes for rechargeable lithium and lithium-ion batteries. Energy Environ. Sci. 2017, 10 (2), 435–459. 10.1039/C6EE02326F.

[ref14] Liu Z.; Liu J.; Zhao S.; Xun S.; Byaruhanga P.; Chen S.; Tang Y.; Zhu T.; Chen H. Low-cost iron trichloride cathode for all-solid-state lithium-ion batteries. Nature Sustainability 2024, 7 (10), n/a 10.1038/s41893-024-01431-6.

[ref15] Kanno R.; Takeda Y.; Takada K.; Yamamoto O. Phase diagram and ionic conductivity of the lithium chloride-iron(II) chloride system. Solid State Ionics 1983, 9–10, 153–156. 10.1016/0167-2738(83)90225-4.

[ref16] Kanno R.; Takeda Y.; Takahashi A.; Yamamoto O.; Suyama R.; Kume S. Structure, ionic conductivity, and phase transformation in new polymorphs of the double chloride spinel, Li2FeCl4. J. Solid State Chem. 1988, 72 (2), 363–375. 10.1016/0022-4596(88)90040-0.

[ref17] Lutz H. D.; Schmidt W.; Haeuseler H. Chloride spinels: A new group of solid lithium electrolytes. J. Phys. Chem. Solids 1981, 42 (4), 287–289. 10.1016/0022-3697(81)90142-6.

[ref18] Lutz H. D.; Pfitzner A.; Cockcroft J. K. Structural Phase Transition and Nonstoichiometry of Li2FeCl4—Neutron Diffraction Studies. J. Solid State Chem. 1993, 107 (1), 245–249. 10.1006/jssc.1993.1344.

[ref19] Wang C.; Hong J. Ionic/Electronic Conducting Characteristics of LiFePO4 Cathode Materials: The Determining Factors for High Rate Performance. Electrochem. Solid-State Lett. 2007, 10 (3), A65 10.1149/1.2409768.

[ref20] Tanibata N.; Takimoto S.; Aizu S.; Takeda H.; Nakayama M. Applying the HSAB design principle to the 3.5 V-class all-solid-state Li-ion batteries with a chloride electrolyte. Journal of Materials Chemistry A 2022, 10 (39), 20756–20760. 10.1039/D2TA05152D.

[ref21] Vinado C. Interfacial Engineering in All Solid-State Lithium Ion Batteries; University of Washington, 2019.

[ref22] Tanibata N.; Kato M.; Takimoto S.; Takeda H.; Nakayama M.; Sumi H. High Formability and Fast Lithium Diffusivity in Metastable Spinel Chloride for Rechargeable All-Solid-State Lithium-Ion Batteries. Advanced Energy and Sustainability Research 2020, 1 (1), 2000025 10.1002/aesr.202000025.

[ref23] Chen H.; Adams S. Bond softness sensitive bond-valence parameters for crystal structure plausibility tests. IUCrJ. 2017, 4 (5), 614–625. 10.1107/S2052252517010211. 28989717 PMC5619853

[ref24] Chen H.; Wong L. L.; Adams S. SoftBV - a software tool for screening the materials genome of inorganic fast ion conductors. Acta Crystallographica Section B 2019, 75 (1), 18–33. 10.1107/S2052520618015718. 32830774

[ref25] Kwak H.; Han D.; Son J. P.; Kim J. S.; Park J.; Nam K.-W.; Kim H.; Jung Y. S. Li+ conduction in aliovalent-substituted monoclinic Li2ZrCl6 for all-solid-state batteries: Li2+xZr1-xMxCl6 (M = In, Sc). Chemical Engineering Journal 2022, 437, 135413 10.1016/j.cej.2022.135413.

[ref26] Liu Z.; Ma S.; Liu J.; Xiong S.; Ma Y.; Chen H. High Ionic Conductivity Achieved in Li3Y(Br3Cl3) Mixed Halide Solid Electrolyte via Promoted Diffusion Pathways and Enhanced Grain Boundary. ACS Energy Letters 2021, 6 (1), 298–304. 10.1021/acsenergylett.0c01690.

[ref27] Wang S.; Bai Q.; Nolan A. M.; Liu Y.; Gong S.; Sun Q.; Mo Y. Lithium Chlorides and Bromides as Promising Solid-State Chemistries for Fast Ion Conductors with Good Electrochemical Stability. Angew. Chem., Int. Ed. 2019, 58 (24), 8039–8043. 10.1002/anie.201901938. 30977261

[ref28] Sang J.; Tang B.; Pan K.; He Y.-B.; Zhou Z. Current Status and Enhancement Strategies for All-Solid-State Lithium Batteries. Accounts of Materials Research 2023, 4 (6), 472–483. 10.1021/accountsmr.2c00229.

[ref29] Ding J.-F.; Zhang Y.-T.; Xu R.; Zhang R.; Xiao Y.; Zhang S.; Bi C.-X.; Tang C.; Xiang R.; Park H. S.; et al. Review on lithium metal anodes towards high energy density batteries. Green Energy & Environment 2023, 8 (6), 1509–1530. 10.1016/j.gee.2022.08.002.

[ref30] Toby B. H.; Von Dreele R. B. GSAS-II: the genesis of a modern open-source all purpose crystallography software package. J. Appl. Crystallogr. 2013, 46 (2), 544–549. 10.1107/S0021889813003531.

[ref31] Momma K.; Izumi F. VESTA: a three-dimensional visualization system for electronic and structural analysis. J. Appl. Crystallogr. 2008, 41, 653 10.1107/S0021889808012016.

