# Supplementary material for: Li2FeCl4 as a Cost-Effective and Durable Cathode for Solid-State Li-Ion Batteries
Source: ACS Energy Lett. 2024 Oct 21;9(11):5464–70. doi: 10.1021/acsenergylett.4c02376 (PMC11555680; doi:10.1021/acsenergylett.4c02376)
Supplement: Supplementary file 1 — nz4c02376_si_001.pdf [file nz4c02376_si_001.pdf]

Supporting Information

Li_2FeCl_4 as a Low-cost and Long-lasting Cathode for Solid-State Li-Ion Batteries

Zhantao Liu[1], Guangxing Zhang[1], Jakub Pepas[1], Yifan Ma[1], Hailong Chen[1]*

1. George W. Woodruff School of Mechanical Engineering, Georgia Institute of Technology; Atlanta, GA, 30332, United States.

Materials synthesis

Li_2FeCl_4 was synthesized via a mechanochemical method, followed by an annealing treatment. LiCl (sigma, 99%) and $FeCl_2$ were weighed in a molar ratio of 2:1 and ball-milled at 500 rpm for 5h in ZrO_2 jars (50 mL) with ZrO_2 balls (diameter: 10 mm) in a planetary ball mill (PM 200, Retsch) followed by annealing at 400 °C. All the processes were carried out under Ar atmosphere.

$Li_3YCl_3Br_3$ and $Li_{2.75}In_{0.75}Zr_{0.25}Cl_6$ were prepared using a similar method. For $Li_3YCl_3Br_3$ synthesis, LiBr and YCl_3 were ball-milled at 500 rpm for 5h followed by sintering at 400 °C. For $Li_{2.75}In_{0.75}Zr_{0.25}Cl_6$ synthesis, LiCl, $InCl_3$ and $ZrCl_4$ were ball-milled at 500 rpm for 5h followed by sintering at 420 °C.

The Li_3YCl_6 was synthesized mechanochemically from LiCl and YCl_3 at 500 rpm for 5h.

Electrochemical measurements

The ionic conductivity was measured using an electrochemical impedance analyzer (VMP3, Bio-logic) and a homemade electrochemical cell. Typically, 0.5~1 g electrolyte powders were cold pressed into pellets with a diameter of ½ inch at a pressure of 294 MPa. Two pieces of Al foils were used as current collectors, and the data was collected at varied temperatures in the frequency range of 1MHz to 1Hz with an AC amplitude of 50 mV.

The solid-state cells were fabricated with a home-made setup. The composite cathode was made by mixing as-synthesized Li_2FeCl_4, $Li_{2.75}In_{0.75}Zr_{0.25}Cl_6$ and carbon black in a weight ratio of 55: 40: 5 in a mortar by hand. InLi alloy was used as anode. 130 mg $Li_{2.75}In_{0.75}Zr_{0.25}Cl_6$[1] was pressed at 294 MPa to form a dense pellet within a PMMA sleeve (ID: ½ inch). 80 mg $Li_3YCl_3Br_3$[2] was used as protective layer against InLi alloy, which was spread on one side of the $Li_{2.75}In_{0.75}Zr_{0.25}Cl_6$

pellet and pressed at 294 MPa. ~10 mg composite cathode was spread on the other side of the $Li_{2.75}In_{0.75}Zr_{0.25}Cl_6$ pellet at pressed at the same pressure. Finally, the anode part was fabricated by attaching a piece of Indium foil on $Li_3YCl_3Br_3$ side and followed by attaching another piece of Lithium metal foil and pressed at ~50 MPa. Galvanostatic charge/discharge was conducted on the LAND battery test system at room temperature.

Ex situ Synchrotron Diffraction Characterization

Synchrotron X-ray diffraction patterns were collected at synchrotron X-ray source at beamline 17-BM at the Advanced Photon Source (APS) and at 28ID-2 beamline of the National Synchrotron Light Source II (NSLS II). Rietveld refinements against the XRD data were performend with using GSAS II[3]. The crystal structure was visualized by VESTA[4].

Operando Synchrotron Diffraction Characterization

Operando synchrotron diffraction measurements were conducted in a homemade solid-state cell at the 28-ID-B beamline at National Synchrotron Light Source II (NSLS II) in Brookhaven National Laboratory. In this cell, single Li_3YCl_6 layer was employed instead of $Li_{2.75}In0_{.75}Zr_{0.25}Cl_6/Li_3YCl_3Br_3$ bilayer as solid electrolytes. X-ray transparent tube with an inner diameter of 1/8 inch was employed as sleeve. ~10 mg Li_3YCl_6 was pressed into a pellet within the tube. The composite cathode was made by mixing as-synthesized Li_2FeCl_4, Li_3YCl_6 and carbon black in a weight ratio of 55:40:5 in a mortar by hand. In this cell, the cathode mass loading was ~2 mg. The diffraction patterns were collected in transmission mode. The incident beam size along vertical direction was narrowed down to ~100 um and the vertical length of the cell was scanned layer-by-layer with a step size of 33 um. The data acquisition time was 60s.

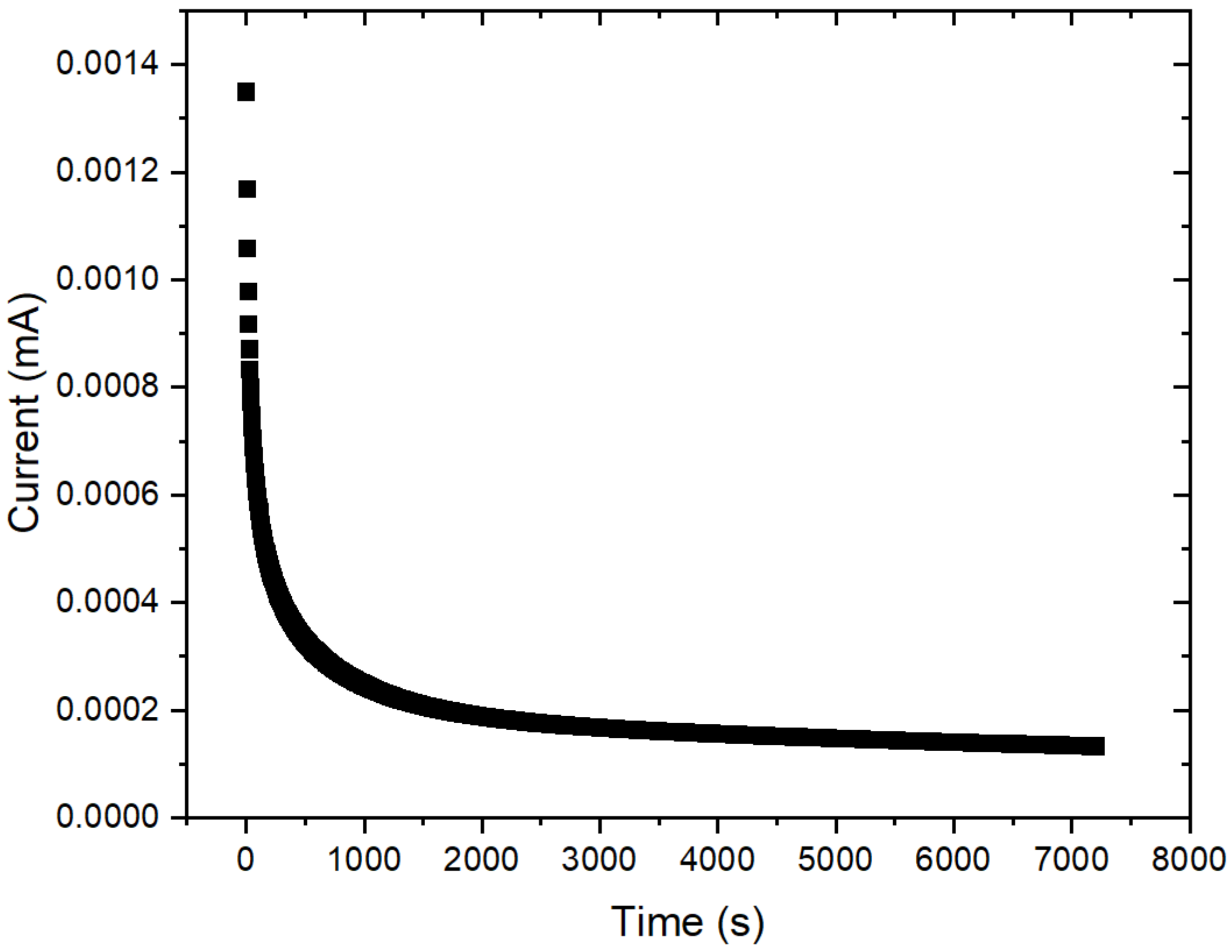

Fig. S1. Electronic conductivity test of Li_2FeCl_4 using symmetric cell configuration with blocking electrodes under a constant voltage of 0.3 V.

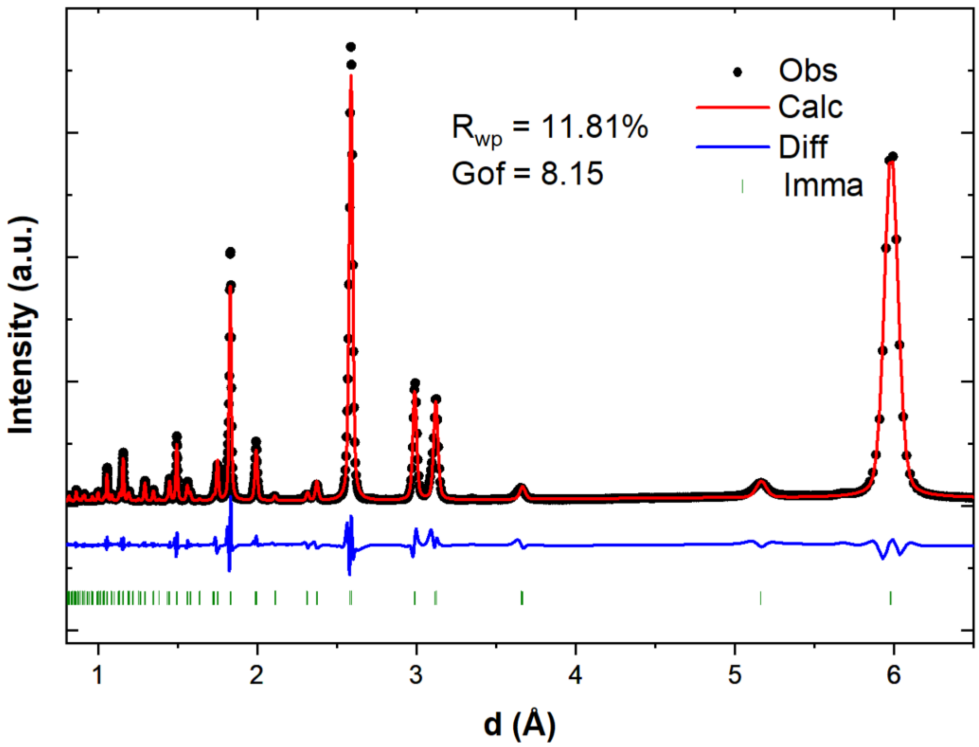

Fig. S2. Rietveld refinement against Li_2FeCl_4 synchrotron powder diffraction pattern using an ordered spinel superstructure (space group: *Imma*).

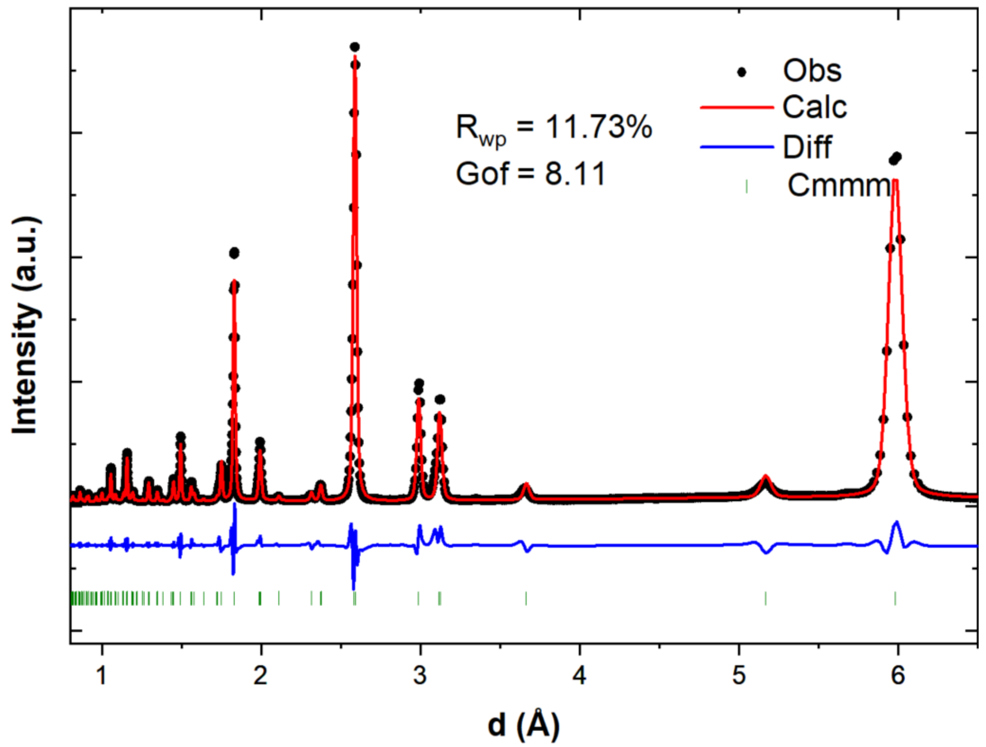

Fig. S3. Rietveld refinement against Li_2FeCl_4 synchrotron powder diffraction pattern using the $SnMn_2S_4$-type NaCl superstructure (space group: *Cmmm*).

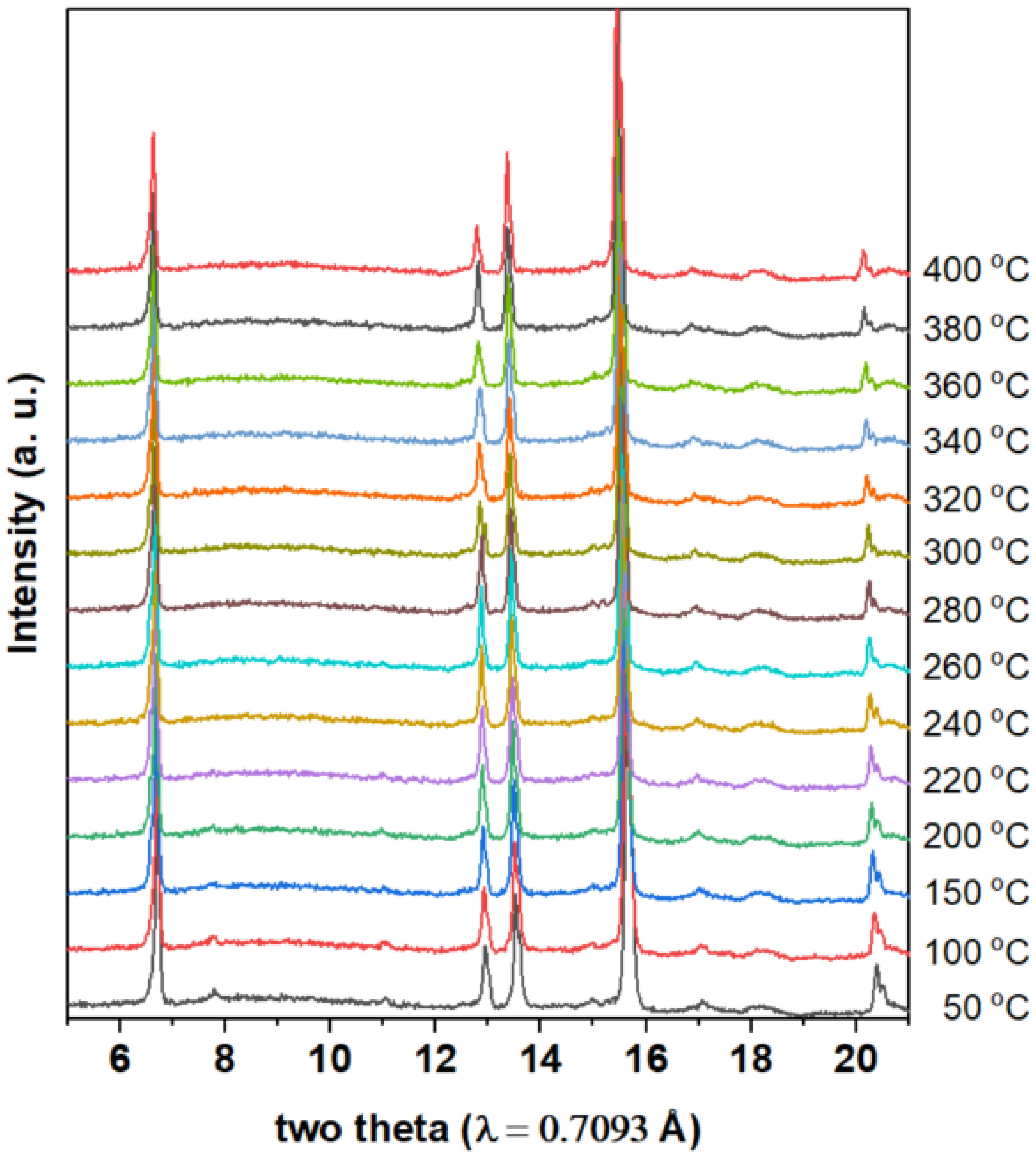

Fig. S4. In situ XRD of Li_2FeCl_4.

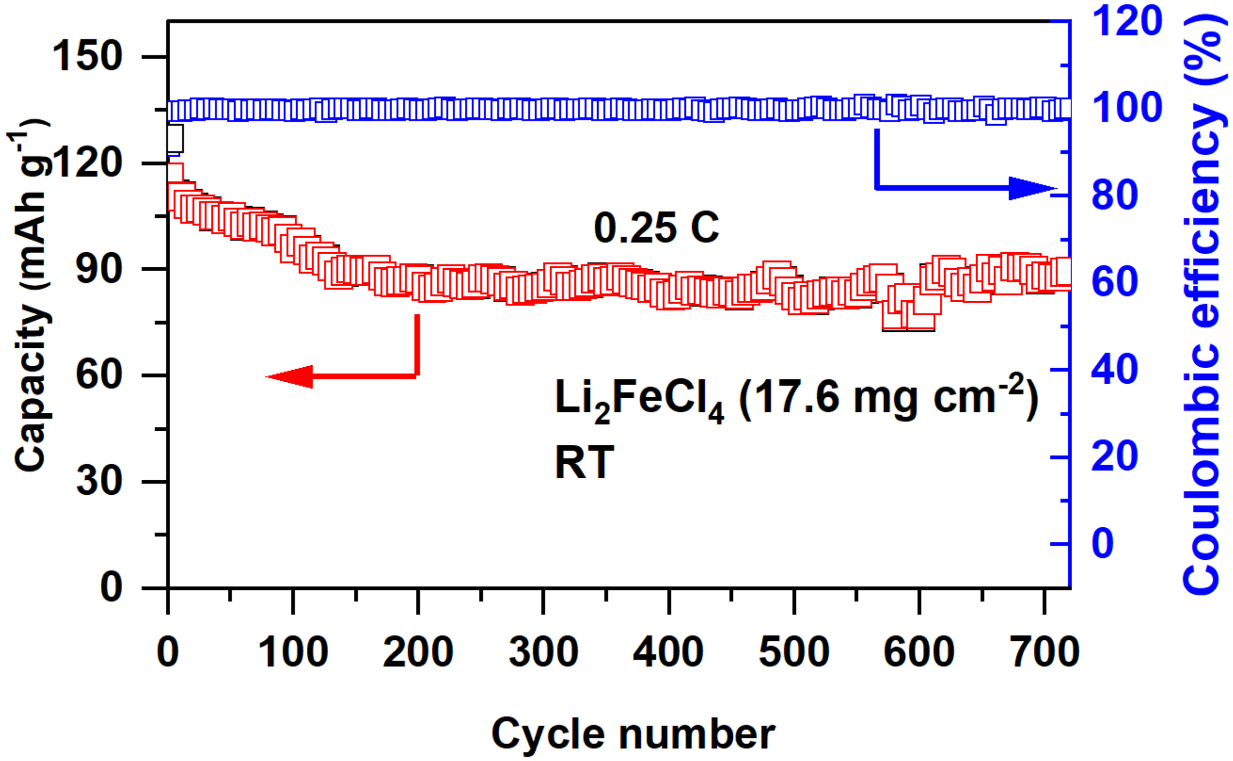

Fig. S5. Electrochemical performance of Li_2FeCl_4 cells with a $FeCl_3$ mass loading of 17.37 mg cm^{-2}.

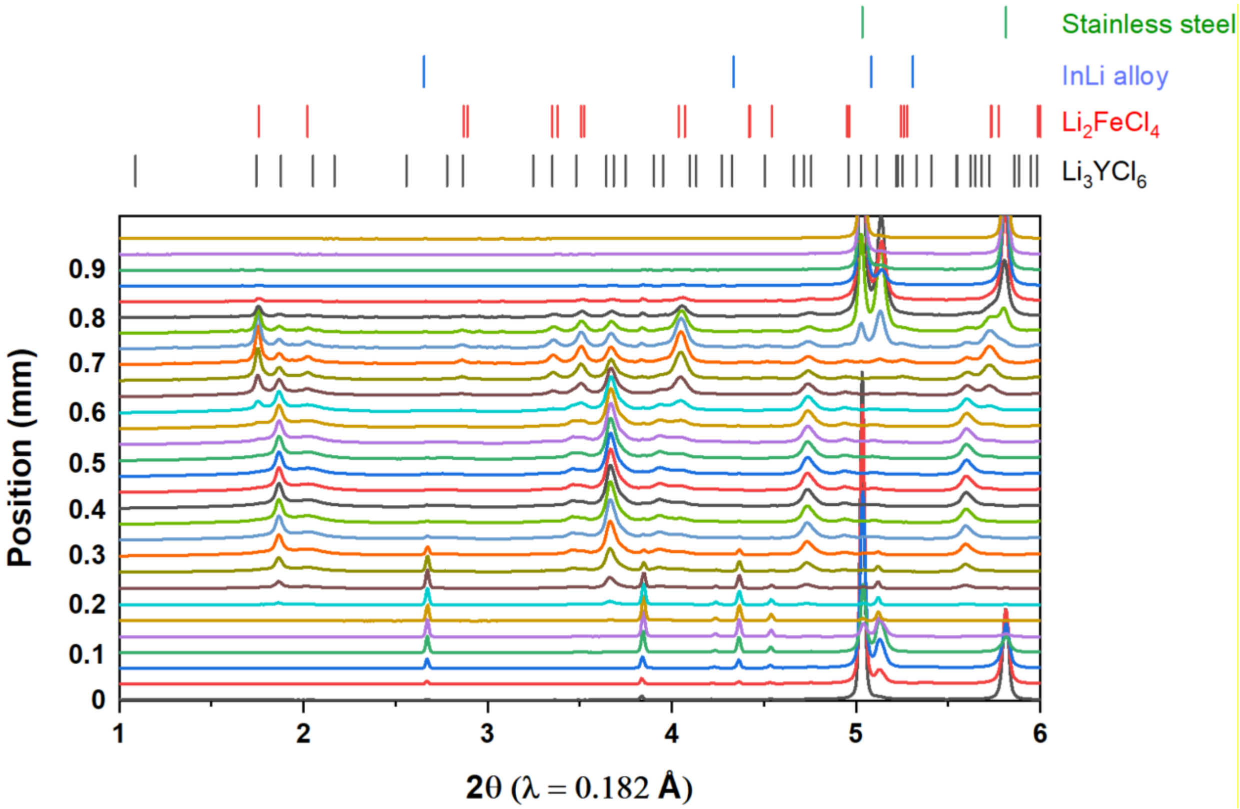

Fig. S6. XRD patterns of the entire Li_2FeCl_4 cell.

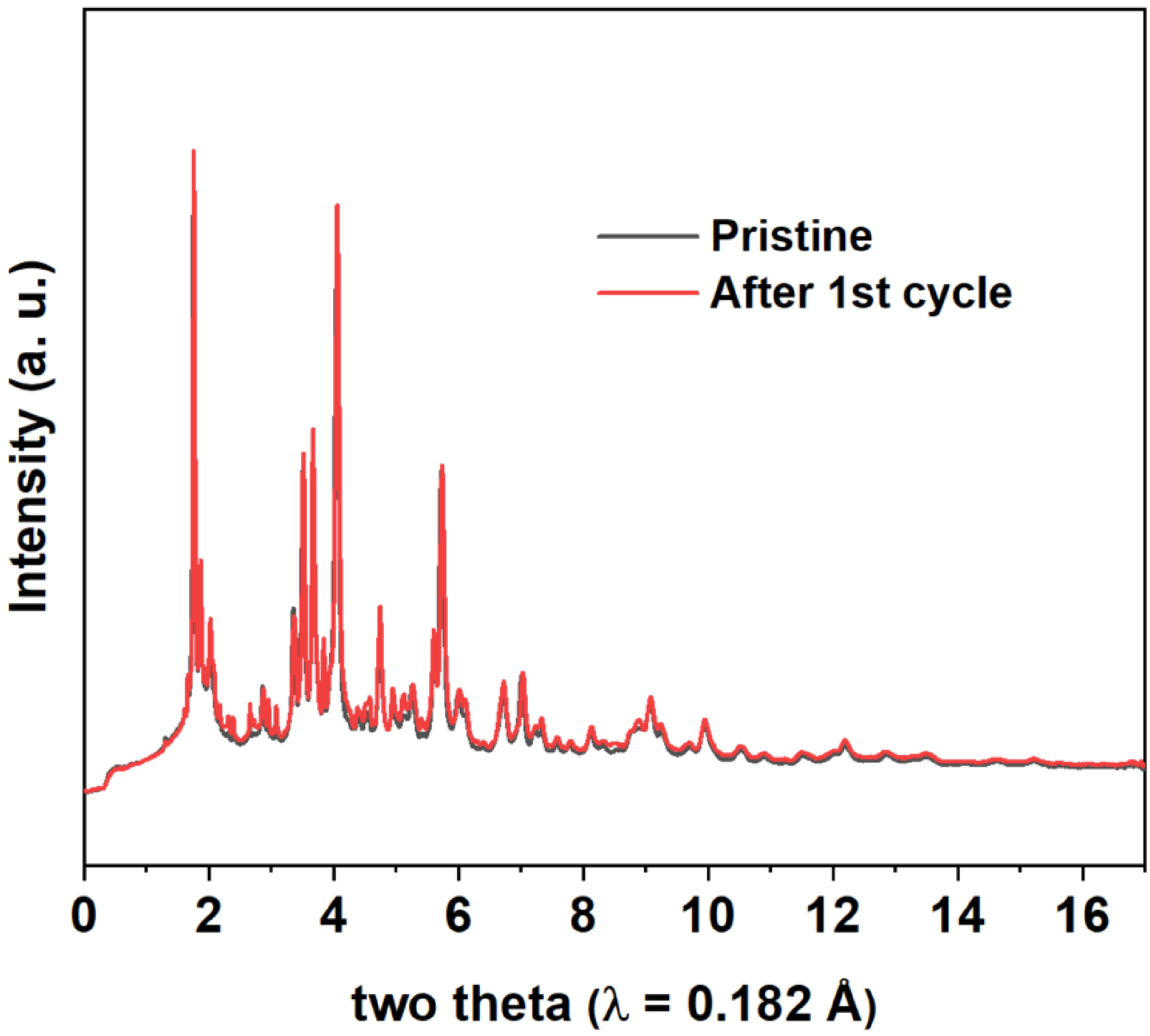

Fig. S7. The comparison of the in situ XRD patterns of Li_2FeCl_4 cathode layer at the pristine state and after one complete cycle.

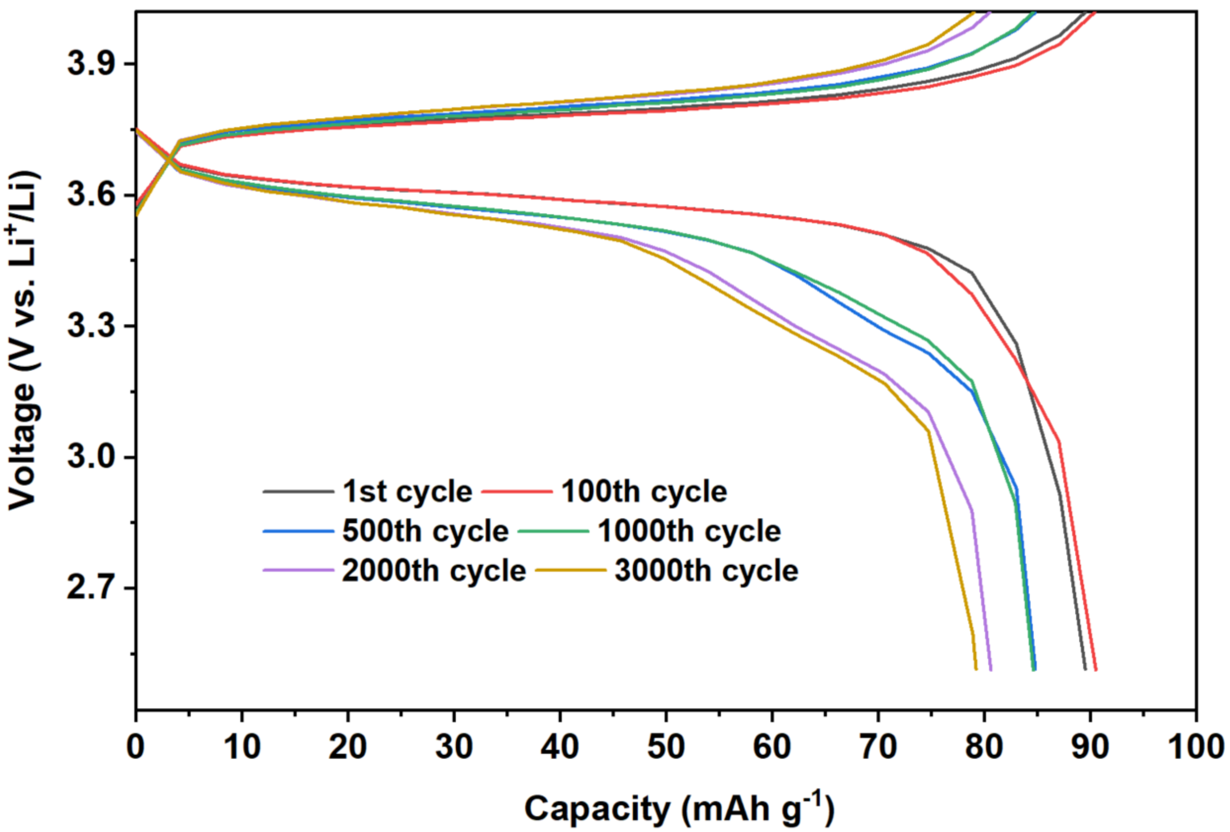

Fig. S8. Comparison of charge/charge curves at different cycle numbers.

Table S1. Refined structure of Li_2FeCl_4 at room temperature using synchrotron diffraction data ($Cmmm$)

S.G. $Cmmm$						
a = 7.324(9) Å	b = 10.34(6) Å	c = 3.664(2) Å	vol = 277.7(0) $Å^3$			
Site	Wyck.	x	y	Z	Occ.	U_{iso} ($Å^2$)
Li1	4f	0.25	0.25	0.5	0.85(3)	0.013(7)
Fe1	4f	0.25	0.25	0.5	0.14(7)	0.013(7)
Li2	2a	0	0	0	0.14(4)	0.02(1)
Fe2	2a	0	0	0	0.85(6)	0.02(1)
Cl1	4h	0.220(4)	0	0.5	0.85(10)	0.007(6)
Cl2	4f	0	0.247(6)	0	1	0.02(3)

References

1. Kwak, H.; Han, D.; Son, J. P.; Kim, J. S.; Park, J.; Nam, K.-W.; Kim, H.; Jung, Y. S., Li+ conduction in aliovalent-substituted monoclinic Li2ZrCl6 for all-solid-state batteries: Li2+xZr1-xMxCl6 (M = In, Sc). *Chemical Engineering Journal* **2022,** *437*, 135413.
2. Liu, Z.; Ma, S.; Liu, J.; Xiong, S.; Ma, Y.; Chen, H., High Ionic Conductivity Achieved in Li3Y(Br3Cl3) Mixed Halide Solid Electrolyte via Promoted Diffusion Pathways and Enhanced Grain Boundary. *ACS Energy Letters* **2021,** *6* (1), 298-304.
3. Toby, B. H.; Von Dreele, R. B., GSAS-II: the genesis of a modern open-source all purpose crystallography software package. *Journal of Applied Crystallography* **2013,** *46* (2), 544-549.
4. Momma, K.; Izumi, F., VESTA: a three-dimensional visualization system for electronic and structural analysis. *J. Appl. Crystallogr.* **2008,** *41*, 653.
